# Methodology and software to detect viral integration site hot-spots

**DOI:** 10.1186/1471-2105-12-367

**Published:** 2011-09-14

**Authors:** Angela P Presson, Namshin Kim, Yan Xiaofei, Irvin SY Chen, Sanggu Kim

**Affiliations:** 1 Department of Biostatistics, University of California Los Angeles, School of Public Health, Los Angeles, CA 90095, USA; 2 UCLA AIDS Institute, University of California Los Angeles, Los Angeles, CA 90095, USA; 3 Korea Research Institute of Bioscience and Biotechnology, 111 Gwahangno, Yuseong-gu, Daejeon 305-806, Korea; 4 Medical Genetics Institute, Cedars-Sinai Medical Center, Los Angeles, CA 90048, USA; 5 Department of Microbiology, Immunology, & Molecular Genetics, University of California Los Angeles, Los Angeles, CA 90095, USA; 6 Department of Medicine, University of California Los Angeles, Los Angeles, CA 90095, USA

## Abstract

**Background:**

Modern gene therapy methods have limited control over where a therapeutic viral vector inserts into the host genome. Vector integration can activate local gene expression, which can cause cancer if the vector inserts near an oncogene. Viral integration hot-spots or 'common insertion sites' (CIS) are scrutinized to evaluate and predict patient safety. CIS are typically defined by a minimum density of insertions (such as 2-4 within a 30-100 kb region), which unfortunately depends on the total number of observed VIS. This is problematic for comparing hot-spot distributions across data sets and patients, where the VIS numbers may vary.

**Results:**

We develop two new methods for defining hot-spots that are relatively independent of data set size. Both methods operate on distributions of VIS across consecutive 1 Mb 'bins' of the genome. The first method 'z-threshold' tallies the number of VIS per bin, converts these counts to z-scores, and applies a threshold to define high density bins. The second method 'BCP' applies a Bayesian change-point model to the z-scores to define hot-spots. The novel hot-spot methods are compared with a conventional CIS method using simulated data sets and data sets from five published human studies, including the X-linked ALD (adrenoleukodystrophy), CGD (chronic granulomatous disease) and SCID-X1 (X-linked severe combined immunodeficiency) trials. The BCP analysis of the human X-linked ALD data for two patients separately (774 and 1627 VIS) and combined (2401 VIS) resulted in 5-6 hot-spots covering 0.17-0.251% of the genome and containing 5.56-7.74% of the total VIS. In comparison, the CIS analysis resulted in 12-110 hot-spots covering 0.018-0.246% of the genome and containing 5.81-22.7% of the VIS, corresponding to a greater number of hot-spots as the data set size increased. Our hot-spot methods enable one to evaluate the extent of VIS clustering, and formally compare data sets in terms of hot-spot overlap. Finally, we show that the BCP hot-spots from the repopulating samples coincide with greater gene and CpG island density than the median genome density.

**Conclusions:**

The z-threshold and BCP methods are useful for comparing hot-spot patterns across data sets of disparate sizes. The methodology and software provided here should enable one to study hot-spot conservation across a variety of VIS data sets and evaluate vector safety for gene therapy trials.

## Background

Gene therapy holds promise for curing HIV, cancer and blood disorders by targeting and altering expression of disease related genes [1-3]. Successful gene therapy relies on the safe and efficient introduction of therapeutic genetic material into the host genome by a modified virus, such as lentivirus (LV) or murine leukemia virus (MLV). Diseases that have been corrected by gene therapy include X-linked severe combined immunodeficiency (X-linked SCID), adenosine deaminase severe combined immunodeficiency (ALD), and X-linked chronic granulomatous disease (CGD) [4-9]. However, the successes of gene therapy have been somewhat offset by the accompanying risk of 'insertional mutagenesis', or activation of local gene expression near the integration site. In the X-linked SCID studies, which employed MLV vectors, 25% of patients developed T-cell lymphoproliferative syndrome within five years post-transplant due to vector insertion near *LMO*2, *BMI*1 and *CCND*2 proto-oncogenes [8,10-12]. While no cancer cases have yet been reported from LV studies, both LV and MLV vector types exhibit preferential integration or integration 'hot-spots'. As a result, it is important to study genes and DNA features located within or near vector integration site (VIS) hot-spots to gain insight into the mechanism of vector integration and predict potential long-term toxicity of gene therapy vectors [13-16].

The non-random nature of viral integration and the potential for integration events to cause toxicity and cancer have long been realized [17,18]. However, a formal VIS hot-spot definition was not described until the turn of the century when Suzuki et al. (2002) developed a definition for retroviral integration in cancer cells to discover potential cancer-related genes [19]. The authors referred to a hot-spot as a Common Insertion Site (CIS) and defined them as ≥4 integrations within a 100 kb region, 3 integrations within 50 kb or 2 within 30 kb. Similar definitions were adopted by others, such as 3 VIS within 100 kb [15] and 4 within 104 kb [20]. As in these examples, it has been useful to tailor the hot-spot definition to the data set under examination. This is most commonly done using computer simulations or mathematical analysis to select a CIS definition relative to the current data set [19,21-23]. Wu et al. (2006) suggested that unselected or acute infection VIS data should be used as a reference or control data set for defining hot-spots in the corresponding post transplant data [21]. The authors reasoned that natural vector-specific biases exist, and for gene therapy applications it is most interesting to see how the post-transplant VIS preferences compare to this reference (rather than for example, randomly selected genomic locations). Thus a suitable threshold for defining hot-spots in post-transduced cells should detect few to no hot-spots in the acute infection data.

More recently, Biffi et al. (2011) proposed a validation step following traditional CIS analysis that confirms CIS significance by comparing integration frequencies among gene transcription units within the CIS interval and its flanking genes [23]. This biologically motivated approach is based on the concept that significant CIS should identify genes with high integration frequencies, as this could reveal potential for insertional mutagenesis.

While effective CIS definitions have been developed for single data set analysis, it is less obvious how to consistently define hot-spots across multiple data sets of varying size. For example, a data set with 4000 VIS is more likely to contain 4 VIS within 100 kb just by chance than a data set with 400 VIS. While one can tailor the CIS definition to data set size, to our knowledge the utility of this approach has not been fully explored. Furthermore, the CIS definition has been developed for MLV data sets, where clustering tends to occur on the kilobase scale. In an accompanying publication we describe LV clustering in rhesus macaque, where some hot-spots appear to span several megabases [23,24]. With these concepts in mind, we developed two new hot-spot definitions based on z-transformed VIS densities. The first method 'z-threshold' simply applies a threshold to z-transformed VIS densities. The second method applies a bayesian change-point analysis 'BCP' to z-transformed VIS densities. BCP models have been applied to other DNA sequence problems including the detection of recombination events and DNA copy number variations [25-29]. Using simulated and real data sets we show that the z-threshold and BCP methods improve over a conventional CIS method by defining hot-spots relatively independent of data set size. The accompanying software implements these definitions and provides graphical tools for visualizing VIS patterns and hot-spots across data sets on the genome and chromosome scales.

## Results and Discussion

**Figure 1:**
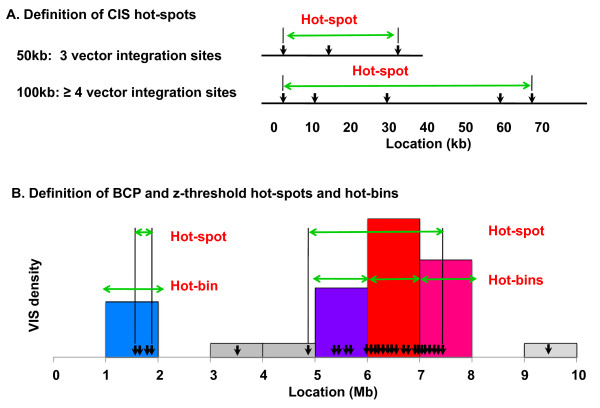
**Definition of CIS, BCP and z-threshold hot-spot methods**. (A) The CIS hot-spot definition implemented here is based on a commonly used density metric [19,21]. (B) Our 'z-threshold' and 'BCP' hot-spot definitions operate on a partition of the genome into 1 Mb bins. The number of VIS per megabase bin is tallied and then converted to a z-score by subtracting the mean and dividing by the standard error, calculated across all bins. Bins with high z-scores are called 'hot-bins'. Hot-spots are defined by grouping consecutive hot-bins and setting each external boundary to the closest VIS.

The fundamental tenets of our hot-spot definitions include 1) hot-spot identification that is relatively independent of data set size, 2) identification of few to no hot-spots in the corresponding acute infection or pre-transplant data, and 3) hot-spot identification and visualization on both the kilobase and megabase scales. We develop two novel methods: z-threshold and BCP, and compare them with a conventional CIS definition: ≥3 VIS within 50 kb and ≥4 VIS within 100 kb (Figure 1A). The z-threshold and BCP methods are useful for studying hot-spot conservation across VIS data sets of varying size, for example data collected on multiple subjects, time-points or cell types. We also describe numerical summary measures that indicate the extent of VIS clustering, and a statistical test for comparing hot-spot conservation across data sets. Methods are illustrated on real data from the X-linked ALD, CGD and X-linked SCID studies [6-9,20] and simulated data derived from the X-linked ALD study (Additional File 1).

**Figure 2:**
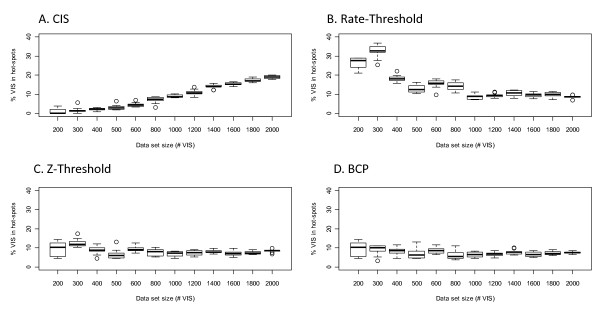
**Comparison of % VIS in hot-spots for CIS, rate-threshold, z-threshold and BCP analyses for data sets with 200-2000 VIS**. Boxplots indicate distributions of % VIS in hot-spots for 12 simulated data sets per data set size for the CIS, rate-threshold, z-threshold and BCP methods. Data set sizes are in increments of 100 for smaller data sets (200-600 VIS) to view performance when less data is available, and in increments of 200 otherwise. The CIS method (A) shows an increasing percentage of VIS in hot-spots with increasing data set size across all data set sizes. The z-threshold (C) and BCP (D) methods give the most consistent % VIS in hot-spots across data set sizes, with both methods giving most consistent results for data sets with ≥300 VIS.

The z-threshold and BCP methods rely on first partitioning the genome or chromosomes into non-overlapping 1 Mb bins. The megabase unit was chosen because it worked well for defining hot-spots in our LV data sets, however other units are certainly possible and may be desirable for other vector types. The number of VIS per bin gives a simple VIS density distribution, and imposing a threshold to define high-count bins as hot-spot regions would be similar to the CIS method. An obvious extension then for comparing hot-spots among data sets with differing VIS numbers, would be to divide the bin counts by the total number of VIS in the data set, and apply a threshold to these bin rates. (Note that rather than defining hot-spots in 1 Mb units, we select the closest VIS to each boundary, see Figure 1B). This 'rate-threshold' approach more uniformly defines hot-spots across data sets of varying size than the CIS method (Figure 2A-B), but it is still affected by data set size (Spearman p-value = 0.001). The threshold for defining the Figure 2B hot-spots corresponded to the 99.92 percentile of the X-linked ALD acute infection data set rates, 0.006. In the following section we show that applying a z-score transformation to the bin counts improves the uniformity of hot-spot results across data sets of varying size.

### Z-threshold hot-spot definition

The z-threshold definition also operates on non-overlapping 1 Mb bins, but in this case the bin counts are standardized by both the overall mean and variance of a data set's bin count distribution. For bin *i*, the z-score *X_i _*is given by: $\left[C_{i}\;\text{ - }\;\bar{C}\right]∕\left[\text{SE}\left(C\right)\right]$, where *C_i _*is the number of VIS in bin *i *and **C **denotes the vector of all *n *megabase bin counts in the genome *C*_1_, *C*_2_, ..., *C_n_*, where chromosomes were artificially 'strung together' to form one continuous genomic sequence. We then impose a threshold of 422, corresponding to the 99.92 percentile of the X-linked ALD acute infection data set z-scores, and define all bins that exceed the threshold as 'hot-bins'. These hot-bin regions can then be further refined by selecting the closest VIS to the bin boundaries (Figure 1B). We refer to these refined hot-bin regions as hot-spots. While hot-spots are the main focus of this paper, hot-bins are a useful approximation that can be used in statistical analyses of hot-spot conservation. Results for evaluating the percentage of VIS in hot-spots across simulated data sets of increasing size show that the z-threshold method is relatively invariant to data set size (Figure 2C, Spearman p-value = 0.140).

### BCP hot-spot definition

The concept of defining highly clustered VIS regions in the genome can also be viewed as a change-point problem. In this framework change-points are genomic locations that delineate between consecutive regions of high and low VIS concentration. We implemented the change-point model using the bcp package in R [26,30,31], operating on the z-scores of the megabase bins described in the previous section. Again we analyzed the complete genome to allow detection of more hot-spots on chromosomes that had a high VIS density relative to other chromosomes. We ran the bcp function with the default values except that we increased the number of iterations from 500 to 10000 to improve convergence (Additional File 2). We defined hot-bins by applying the same threshold to the posterior means. Similar to the z-threshold method the hot-spots are relatively uniform across the simulated data sets (Figure 2D, Spearman p-value = 0.099), however both methods give more consistent results for data sets with ≥ 300 VIS.

#### Details of the Bayesian change-point model

We applied a Bayesian change-point analysis to the bin z-scores using a Gaussian model, which determined the posterior probability of a change in z-scores at each bin as well as the posterior means of the bins [32]. Briefly, we define the z-score of bin *i *as *X_i_*, for *i *= 1, ..., *n *where *X_i _~ *N(*μ_i_*, σ^2^); *μ *= (*μ*_1_, *μ*_2_, ..., *μ_n_*); and define an unknown partition of the *n *bins into *b *blocks as **B **= (*B*_1_, *B*_2_, ..., *B_b_*) where *B_i _*= 0, 1 such that '1' indicates a change-point. Define *μ_ij _*as the average z-score of the (*i *+ 1, *j*) block, so that $X_{ij}\sim \text{N}\left(\mu_{0},\sigma_{0}^{2}∕\left(j-i\right)\right)$. The likelihood of the data is given by

$$L\left(X|B,\mu_{0},w\right)\propto \frac{w^{b∕2}}{{\left[WSS+BSSw+wn{\left(\mu_{0}-\bar{X}\right)}^{2}\right]}^{n∕2}}$$

where *w *is the ratio of the error variance to the total variance, and *WSS *and *BSS *are the within and between block sum of squares, respectively [31,32]. On each MCMC iteration, a change-point status *B_i _*is sampled for each position *i *according to probability Pr(*B_i _*= 1|**X**, *B_j_*, *j ≠ i*)/Pr(*B_i _*= 0|**X**, *B_j_*, *j *≠ *i*). After *N *iterations, the set of partitions **B_1_**, **B_2_**, ..., **B_N _**were averaged to obtain the posterior probabilities *P*_1_, *P*_2_, ..., *P*_*n *- 1 _of a change-point at each bin. We defined high density or hot-bins by applying a threshold to the posterior bin means *μ*_1_, *μ*_2_, ..., *μ_n_*. We also used the average of the change-point probabilities $\bar{P}$ to evaluate the extent of clustering in a data set.

### Results for human ALD, X-linked SCID, and CGD data analysis

**Figure 3:**
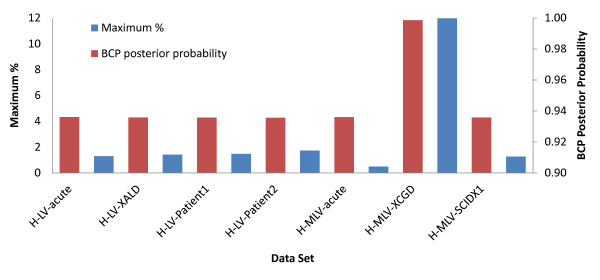
**Comparison of VIS clustering among data sets**. We developed two methods to describe the extent of VIS clustering. The first method 'maximum %' is simply the maximum bin's z-score divided by the total number of VIS in the data set, $100\cdot \max\left(X\right)∕\overset{n}{\underset{i=1}{\sum }}C_{i}$. Data sets with a maximum % > 8 indicate a high degree of clustering. The second method 'BCP posterior probability' is calculated after running the Bayesian change-point analysis, and is simply one minus the average of the posterior probabilities of a change point occurring at each bin, $1-\bar{P}$. BCP posterior probabilities > 0.98 indicate a high degree of clustering. Both methods indicate that the CGD data exhibits a high degree of clustering with a maximum % and BCP posterior probabilities of 11.98 and 0.999, respectively, in comparison to the other data sets which ranged from 0.5-1.48 and 0.9356-0.9361, respectively.

We analyzed seven LV and MLV data sets from five different human VIS studies (Table 1). Figure 3 gives an overview of the relative VIS clustering in these data sets using one minus the average of the change-point probabilities $1-\bar{P}$, and the maximum z-score divided by the number of VIS, $100\cdot \text{max}\left(X\right)∕\sum_{i=1}^{n}C_{i}$. The CGD study data exhibits the highest degree of clustering according to both measures, and the MLV acute infection data exhibits the least clustering according to the maximum z-score method.

**Table 1:** Descriptions of human lentivirus (LV) and murine leukemia virus (MLV) vector integration site (VIS) data sets.

Data set Name	Study	#	Type	# VIS	Time points	Cell Type
H-LV-XALD	X-Linked ALD [8]	2	LV	2401	6-24 m	CD:34,15,3,14,16,19,56, LM
H-LV-Patient1	X-Linked ALD [8]	1	LV	1627	6-24 m	CD:34,15,3,14,16,19,56, LM
H-LV-Patient2	X-Linked ALD [8]	1	LV	774	6-20 m	CD:34,15,3,14,16,19
H-LV-acute	X-Linked ALD [8]	pre	LV	922	--	CD34
H-MLV-XCGD	CGD [9]	2	MLV	384	1-45 m	CD:3,14,15,19; PB, BM, G
H-MLV-XSCID	SCIDX1 [6,7]	14	MLV	864	4-41 m	CD:3,13,14,19; PBL, PBMC
--	SCIDX1 [6]	5	MLV	303	9-30 m	CD:3, 13
--	SCIDX1 [7]	9	MLV	561	4-41 m	CD:3,14,19; PBL, PBMC
H-MLV-acute	GFP [20]	pre	MLV	1398	1-12 days PT	CD34

The seven data sets analyzed here originated from five LV and MLV studies. Column one indicates the data set name given in this paper, and the 'Study' column indicates the parent study where X-linked ALD (adrenoleukodystrophy), CGD (chronic granulomatous disease) and SCIDX1 (X-linked severe combined immunodeficiency) refer to human clinical trials, and GFP (green fluorescent protein) indicates a control vector study. The H-MLV-XSCID data set was derived from two different studies [6,7] as indicated. The '#' column indicates the number of subjects analyzed; 'Type' refers to vector type, LV or MLV; '# VIS' indicates the number of unique vector integration sites; 'Time points' indicates the number of time points from which samples were obtained; and 'Cell Type' indicates the cells from which VIS were derived. LAM sequencing was used exclusively in all studies except the GFP study which used an LM/LAM combination.

For both the simulated and real data sets analyzed here, as well as the real data sets analyzed in our accompanying publication [24], the BCP and z-threshold hot-spot results were similar with a few exceptions. The BCP method detected fewer hot-spots in the acute infection data sets. For example, the BCP method detected no hot-spots in the H-MLV-acute data, while two hot-spots were detected by the z-threshold method for this data set. In some cases, the BCP method can accentuate signals that are both strong and sustained by picking up lower signal bins adjacent to the strong signal (Additional File 3). It can also miss short signals that are near the cut-off threshold. In the highly clustered CGD data set the BCP method was unable to detect a short but strong signal on chromosome 3. While the BCP method is designed to detect short but strong signals and weak but sustained signals [32], in practice it can miss signals in sparse data sets or data sets with only a few strong but short signals. As a result, we have set 300 as the lower bound for data set size using the BCP approach, and smaller data sets with (100,300] VIS are analyzed by the z-threshold method. Furthermore, highly clustered data sets ($1-\bar{P}>0.98$) are analyzed with the z-threshold method. These rules govern the BCP hot-spot results presented in the following sections, where only the CGD data set qualified as highly clustered and all data sets had VIS numbers greater than 300. We recommend that users run both the BCP and z-threshold analyses. Differences could be resolved by either choosing the preferred method based on a visual assessment or taking the union of their results. Due to the similarities between the BCP and z-threshold hot-spot results, for simplicity the following sections compare only the BCP results with the CIS method.

#### BCP hot-spot results

**Figure 4:**
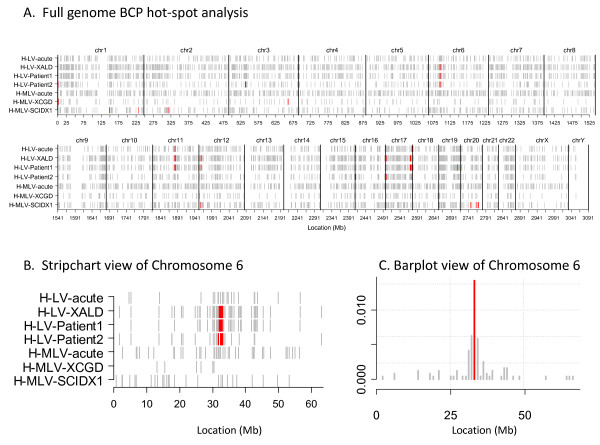
**Graphical displays of BCP hot-spot results from the SCIDX1, CGD, and X-linked ALD trials**. Our hot-spot software produces three types of plots for viewing hot-spot results, (A) a stripchart that displays the full genome for all data sets analyzed, (B) a stripchart that displays results for all data sets, one chromosome at a time; and (C) a barplot that displays results for one data set and chromosome at a time. In all plot types the grey color corresponds to VIS (A, B) or VIS bins (C) that were not defined as hot-spots. In all three plot types the x-axis corresponds to location in megabase units. In plot type C, the y-axis corresponds to bin rate (# VIS per bin/total # VIS) rather than z-score for visual clarity since z-scores can be negative. Color definitions were assigned to each data set independently based on quantiles of its non-zero z-score distribution (ie, the distribution of bin z-scores among bins with non-negative scores). VIS that were located in hot-spot regions corresponding to bins with z-score distributions ≤ 85th percentile are colored light blue, > 85 and ≤ 95 are dark blue, > 95 and ≤ 97.5 are purple, > 97.5 and ≤99 are pink and > 99 are colored red. The plots illustrate hot-spots on chromosomes 6, 11, 12 and 17 in the X-linked ALD data set, and the presence of the chromosome 6 hot-spot in both patients analyzed separately. The MLV data sets exhibit unique VIS patterns that differ from each other as well as the LV data.

Hot-spot results for the seven data sets are provided in Table 2. Consistent with the cluster results from Figure 3, the CGD data set (H-MLV-XCGD) has the highest median hot-spot density according to both the BCP and CIS methods. The percentage of VIS in BCP hot-spots was similar for the full ALD data set and in patients 1 and 2 considered separately, at 6.21%, 7.74% and 5.56%, respectively. In comparison, the CIS analysis resulted in 22.7%, 15.86% and 5.81%, respectively, where the decreasing percentage of VIS corresponded to decreasing data set size (2401, 1627 and 774 VIS). Figure 4A shows that major hot-spots in the ALD data sets (H-LV-XALD, H-LV-Patient1 and H-LV-Patient2) occurred on chromosomes 6, 11, 12 and 17, and Figures 4B-C show chromosome views of the major hot-spot on chromosome 6. An analogous genome-scale view for the CIS hot-spots can be found in Additional File 4.

**Table 2:** Hot-spot summaries for human LV and MLV data sets using the BCP and CIS hot-spot definitions.

		# Hot-Spots		% VIS in HS		% Coverage		Size (Mb)		% Density*	
**Data Set**	**Total VIS**	**BCP**	**CIS**	**BCP**	**CIS**	**BCP**	**CIS**	**BCP**	**CIS**	**BCP**	**CIS**

H-LV-acute	922	3	11	2.93	5.1	0.101	0.02	1.045	0.055	0.93	8.98
H-LV-XALD	2401	5	110	6.21	22.7	0.213	0.246	1.316	0.069	0.86	2.95
H-LV-Patient1	1627	6	56	7.74	15.86	0.251	0.119	1.291	0.066	0.96	4.69
H-LV-Patient2	774	6	12	5.56	5.81	0.17	0.018	0.874	0.047	1.04	10.07
H-MLV-acute	1398	0	15	0	3.93	0	0.017	0	0.035	0	7.29
H-MLV-XCGD	384	2	3	16.93	16.67	0.037	0.009	0.564	0.09	165.32	228.5
H-MLV-SCIDX1	864	7	22	5.9	11	0.045	0.029	0.197	0.041	4.02	13.82

Relative to the CIS method, the BCP hot-spot definition identified fewer hot-spots containing a smaller percentage of VIS for all data sets. It also gave the most consistent results across the full X-linked ALD data set, and patients 1 and 2. The % VIS in hot-spots (HS) ranged from 5.56% to 7.74% for the BCP method and 5.81% to 22.7% using the CIS hot-spot definition. The CIS definition found similar hot-spot numbers and % VIS in hot-spots for the acute infection data set and post-transplant data for patient 2. Both the BCP and CIS methods indicate differences in hot-spot patterns between the LV and MLV data sets. In particular, the CGD study data had very few hot-spots with high VIS densities. % Density was calculated as (Median # VIS per HS/# Total VIS)*100/Mb. Data set names are as in Table 1.

**Figure 5:**
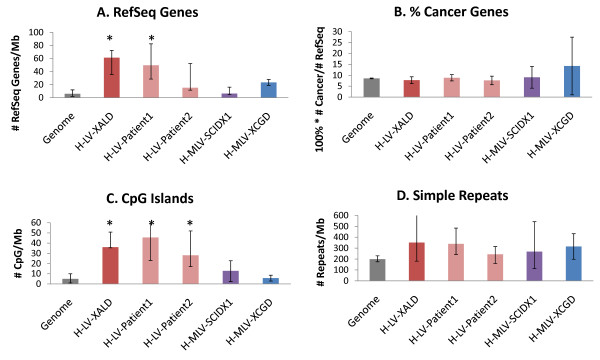
**Genome features of BCP hot-spots in the SCIDX1, CGD, and X-linked ALD trials**. Plots (A), (C), and (D) show the median feature density per Mb and the interquartile range of BCP hot-spot regions in comparison to the genome median. In plot B the enrichment of cancer genes was calculated relative to the RefSeq gene numbers in order to control for gene density differences. The LV data sets showed enrichment of (A) RefSeq genes and (C) CpG islands relative to the genome median (indicated by an asterisk *). No other comparisons to the genome median reached significance at the Bonferroni-corrected level. Overlap of interquartile ranges among the LV data sets shows that their BCP hot-spots have similar genomic features.

While most of the biological discussion of these hot-spots has been relegated to our accompanying publication [24], Figure 5 shows results for commonly studied genomic features including densities of all RefSeq genes, cancer genes, CpG islands and simple repeats. RefSeq gene (5A) and CpG island (5C) densities are higher in the H-LV-XALD, H-LV-Patient1 and H-LV-Patient2 data sets than the genome average. The percentage of genes implicated in cancer is highest in the CGD data set but with only one cancer-related gene *EV I*1 out of seven RefSeq genes, it did not achieve significance. Overlap of interquartile ranges for all genomic features indicates hot-spot similarities for the ALD study and patients 1 and 2.

#### BCP hot-spot conservation

The BCP and z-threshold hot-spot definitions also enable us to numerically compare hot-spot conservation between data sets. This can be done using a Fisher's exact test of hot-bin overlap between a pair of data sets. Previously we described hot-spot overlap for patients 1 and 2 in comparison to the full X-linked ALD data set (Table 3). If we wish to formally test for hot-spot conservation between the patient 1 data set and the X-linked ALD data set, it is useful to use hot-bins rather than hot-spots to control for hot-spot size. Patient 1 has 8 hot-bins and the full X-linked ALD data set has 7 hot-bins (reflecting the decomposition of the Chr 11 hot-spot into three 1 Mb bins), and 6 hot-bins overlap between these data sets. The overlap between these data sets can be summarized in a 2 × 2 table as follows:

**Table 3:** BCP hot-spots for the LV acute infection, full X-linked ALD, and patient 1 and 2 data sets.

Data set	# VIS	Chr	Location (Mb)	# VIS (%)	Size (Mb)	% Density
H-LV-acute	922	Chr 11	65.020-65.721	12 (1.30%)	0.7	1.86
H-LV-acute	922	Chr 16	0.425-1.929	7 (0.76%)	1.5	0.51
H-LV-acute	922	Chr 17	77.185-78.117	8 (0.87%)	0.93	0.93
Median	-	-	-	8 (0.87%)	0.93	0.93

H-LV-XALD	2401	Chr 6	31.967-33.000	35 (1.46%)	1.03	1.41
H-LV-XALD	2401	Chr 11	64.005-66.969	61 (2.54%)	2.96	0.86
H-LV-XALD	2401	Chr 12	6.085-6.993	16 (0.67%)	0.91	0.73
H-LV-XALD	2401	Chr 17	2.000-2.522	17 (0.71%)	0.52	1.36
H-LV-XALD	2401	Chr 17	73.098-74.252	20 (0.83%)	1.15	0.72
Median	-	-	-	20 (0.83%)	1.03	0.86

H-Patient1	1627	Chr 6	31.967-33.000	25 (1.54%)	1.03	1.49
H-Patient1	1627	Chr 11	64.019-66.969	47 (2.89%)	2.95	0.98
H-Patient1	1627	Chr 12	6.085-6.993	14 (0.86%)	0.91	0.95
H-Patient1	1627	Chr 17	73.098-74.252	15 (0.92%)	1.15	0.8
H-Patient1	1627	Chr 17	77.093-77.863	13 (0.8%)	0.77	1.04
H-Patient1	1627	Chr 19	53.882-54.813	12 (0.74%)	0.93	0.79
Median	-	-	-	14.5 (0.89%)	0.98	0.965

H-Patient2	774	Chr 1	2.038-2.954	6 (0.78%)	0.92	0.85
H-Patient2	774	Chr 3	47.057-47.926	6 (0.78%)	0.87	0.89
H-Patient2	774	Chr 6	31.867-32.976	11 (1.42%)	1.11	1.28
H-Patient2	774	Chr 16	0.592-1.838	7 (0.9%)	1.25	0.73
H-Patient2	774	Chr 17	2.000-2.452	7 (0.9%)	0.45	2
H-Patient2	774	Chr 17	76.215-76.864	6 (0.78%)	0.65	1.19
Median	-	-	-	6.5 (0.84%)	0.89	1.04

Five hot-spots were identified by the BCP method for the post infection X-linked ALD data set, ranging in size from 0.52-2.96 Mb with a median density of 0.86% total VIS/Mb. When the ALD study patients were analyzed separately, six hot-spots were observed for each patient with similar sizes and densities. Patient 1 had four hot-spots that overlapped with the full X-linked ALD data set on chromosomes (Chr) 6, 11, 12 and 17; as well as two additional hot-spots on Chr 17 and 19. Patient 2 had two hot-spots that overlapped with the full data set on Chr 6 and 17, as well as four additional hot-spots on Chr 1, 3, 16 and 17. In comparison, the acute infection data with three hot-spots had one overlap with the full ALD data set on Chr 11, a unique hot-spot on Chr 16, and a hot-spot on Chr 17 that overlapped with Patient 1's Chr 17 hot-spot. % Density was calculated as (# VIS per HS/# Total VIS)*100/Mb. Data set names are as in Table 1.

$$\begin{array}{ccc} & \text{H - LV - XALD + } & \text{H - LV - XALD - }\\ \text{H - LV - Patient1 + } & \text{6} & 2\\ \text{H - LV - Patient1 - } & 1 & 3082 \end{array}$$

where the '+' denotes hot-bins and '-' denotes non-hot-bins, and the total number of megabase bins in the human genome is 3091. The Fisher's exact test p-value for this table is < 2.2 × 10^-16^. Patient 2 with 6 hot-bins total and two overlapping with the H-LV-XALD data set is also significant, p-value = 6.6 × 10^-5^. In comparison, the p-value for comparing the H-LV-XALD data set to the acute infection data (with one overlapping hot-bin) is 0.007. In the case where no hot-spots are found in the acute infection data, it might be useful to apply a conservative threshold such as 0.007 for evaluating significance.

### Software for hot-spot analysis

The accompanying R software and tutorial in Additional File 5 enable the user to implement all three hot-spot definitions CIS, BCP and z-threshold for both human and rhesus macaque VIS data sets. The main code file "main.r" is broken into seven major sections listed below, where only the first two are needed to define hot-spots and the remaining code supports additional analyses described in this paper. Run times for each step are provided in parentheses based on an analysis for seven real human data sets with 2401, 1627, 1398, 922, 864, 774, and 384 VIS (or 1196 VIS on average per data set) clocked on a windows machine with an Intel Core i5 2.53 GHz processor and 4 GB of RAM.

1. Install R packages, read in data files, set run parameters. (17.61 sec)

2. Define hot-bins and hot-spots. (12.87 sec for z-threshold, 17.36 min for BCP)

3. Run conventional CIS analysis. (9.97 sec)

4. Get genes overlapping or located within hot-spots. (4.51 min no CIS, 9.28 min with CIS)

5. Compare hot-bins between two groups. (3.10 sec)

6. Statistics for hot-spot gene enrichment. (6.46 min)

7. Merge hot-spot results from two different genome partitions. (1.40 sec)

Steps 2 (for the BCP method only), 4 and 6 are the most time-consuming ranging from 4.51-17.36 minutes for this 7 data set analysis. The BCP method takes considerably longer than the z-threshold approach because the BCP analysis requires 10,000 MCMC iterations (about 2.2 minutes) per data set. In comparison the z-threshold method simply applies a threshold to the bin scores, which happens instantaneously (the additional time for both the BCP and z-threshold methods is spent retrieving hot-spot statistics and plots).

The "main.r" and the "README.txt" files provide guidelines for how to change parameters that govern the CIS, BCP and z-threshold hot-spot methods, such as how to change the threshold for defining hot-bins for the BCP and z-threshold methods (step 1). The default threshold 422, corresponds to the 99.92 percentile of the X-linked ALD acute infection data set z-scores, and was determined using both the data analyzed here and in our accompanying publication [24]. We used this same threshold for the MLV data to compare the LV and MLV vector types. While it works well for a variety of data sets, this threshold can easily be adjusted, for example to the 99.92 percentile (or other percentiles) of the acute infection data in other studies. We provide a simple function *getThreshold *to tailor this threshold to other VIS studies.

Step 2 executes the detection of hot-bins and hot-spots according to the methods outlined in step 1. Step 3 enables the user to run a conventional CIS analysis using either the default definition provided (≥ 3 VIS within 50 kb and ≥ 4 VIS within 100 kb) or alternative definitions as desired. Step 4 finds genes that overlap or are located within hot-spots and/or contain at least one VIS, and step 6 evaluates the significance of hot-spot gene enrichment relative to the genome. Step 5 conducts the analysis described in the "BCP hot-spot conservation" section of this paper, where hot-bins are compared between two groups using a Fisher's exact test. Step 7 enables the user to merge hot-spot results from two different genome partitions, the original partition (bin1 = 0-1 Mb, bin2 = 1-2 Mb,..., bin3091 = 3090-3091 Mb) and an alternative partition that is shifted by a specified amount, such as 0.5 Mb from the original partition (bin1 = 0-0.5 Mb, bin2 = 0.5-1.5 Mb,..., bin3115 = 3090.5-3091.0 Mb). The shift amount [0-1 Mb) is governed by *shiftBins *in step 1, where the original partition is obtained with no shift *shiftBins *= 0.

We allow the user to run alternative partitions of the genome because an anonymous reviewer pointed out that hot-spot results may depend upon the partition. Indeed, when we run the BCP hot-spot analysis with the bins maximally shifted by 0.5 Mb for the H-LV-XALD, H-LV-Patient1 and H-LV-Patient2 data sets there are differences in hot-spot results. These three data sets had 5, 6 and 6 hot-spots for the original bin positions and 6, 5 and 7 hot-spots, respectively for the +0.5 Mb bin positions. There were 4, 3 and 3 matches between the resulting hot-spots from these two partitions for each data set, respectively, or 50-80% correspondence (# matches/# original hot-spots). The merged results yielded 7, 8 and 10 hot-spots, where the overlap between the H-LV-XALD and H-LV-Patient2 hot-spots increased by over 30% from 2/5 to 5/7 (# overlaps/# hot-spots in H-LV-XALD). The overlap between the H-LV-XALD and H-LV-Patient1 hot-spots were similar at 4/5 for the original partition and 5/7 after merging the two partitions (9% decrease). In comparison, the overlap between H-LV-acute and H-LV-XALD also changed little with 1/5 overlaps for the original partition and 1/7 after merging (6% decrease). If we consider four genome partitions (*shiftBins *= 0, 0.25, 0.5 and 0.75) the merged results yield 8, 10 and 11 hot-spots, where the H-LV-XALD and patient data sets see an increase of 16.5% to 7/8 hot-spot overlaps, and the overlap between H-LV-XALD and H-LV-acute increases by 11% to 2/8. Considering additional genome partitions increases the hot-spot yield and improves correspondence between data sets that have similar VIS patterns. As a result, we allow the user to run multiple genome partitions as desired (using the *shiftBins *parameter in step 1) and merge the results (step 7) to achieve a more comprehensive set of hot-spots.

## Conclusions

Lentiviral vectors exhibit strong preferences for specific genomic regions that can encompass several megabases. We describe two novel methods, BCP and z-threshold, that can consistently define hot-spots on the kilobase and megabase scales across data sets of varying size. The BCP method identifies similar numbers of hot-spots 5-6 containing similar percentages of VIS 5.56-7.74% across individual patients and the combined data from the X-linked ALD clinical trial, which varied several fold in size. In comparison, a conventional CIS method identified 12-110 hot-spots containing VIS percentages 5.81-22.7% across the same data sets.

The proposed methods are useful for identifying VIS hot-spots on the megabase scale and comparing genome-wide VIS patterns among data sets of varying size. VIS hot-spot analysis can provide insight into mechanisms of vector integration that will help evaluate the safety of potential gene therapy vectors. The accompanying software and R tutorial will facilitate application of these methods to additional VIS data sets.

## Methods

### Common Insertion Site (CIS) hot-spot definition

While the exact CIS definition varies by study, hot-spots are defined using density thresholds such as 2-4 VIS within a 30-100 kb region [19,21]. Here we adopt the following CIS hot-spot definition: ≥ 3 in 50 kb and ≥ 4 in 100 kb (Figure 1A). To implement the CIS method for an *X*kb window and minimum VIS count *Y*, start at the first position on chromosome and count the number of VIS within the *X*kb window. If the number is ≥ *Y *, the region is considered a hot-spot. Shift the window 1 bp and repeat. Continue until all possible *X*kb windows have been considered for all chromosomes.

### Simulated data analysis

Simulated data sets were constructed by sampling without replacement (ie no VIS was sampled more than once per test data set) from the full human X-linked ALD data set [8], which consisted of 2401 total unique VIS. Simulated data sets consisted of the following sizes: 200, 300, 400, 500, 600, 800, 1000, 1200, 1400, 1600, 1800 and 2000 VIS. Each data set size was sampled 10 times resulting in 120 total simulated data sets. Additional File 1 shows the ranges in numbers of VIS observed per chromosome for the 400 and 1800 VIS data sets in comparison to the actual percentage of VIS per chromosome in the full X-linked ALD data set. The simulated data is provided with the R software in Additional File 5. We tested for a relationship between the percentage of VIS in hot-spots versus data set size by calculating the median percentage of VIS in hot-spots for each size, and performing a Spearman correlation test with the number of VIS per data set.

### Genome feature analysis of BCP hot-spots

RefSeq genes, CpG island and simple repeat data were downloaded from the UCSC genome browser http://hgdownload.cse.ucsc.edu/goldenPath/hg18/database/. Megabase densities of these features were calculated for each BCP hot-spot by dividing the number of features in the hot-spot by the hot-spot size. Median hot-spot densities and their interquartile ranges are plotted in Figure 5. A Wilcoxon rank sum test with continuity correction was used to compare the median density of each data set with the genome median. This resulted in a total of 20 tests (5 data set comparisons × 4 genomic features), so we use a Bonferroni-corrected level of 0.0025 to assess significance. Cancer gene data was obtained from the National Cancer Institute http://ncicb.nci.nih.gov/projects/cgdcp. The percentage of cancer genes was calculated for each data set by dividing the number of cancer genes located in hot-spots by the total number of RefSeq genes located in hot-spots, in order to control for gene density differences.

## Authors' contributions

APP wrote the manuscript and developed the statistical methodology and software. SK and NK retrieved and formatted the VIS data sets. Bioinformatics analyses were conducted by NK, SK, YX and APP. ISYC provided biological direction. All authors read and approved the final manuscript.

## Authors' information

ISYC has a financial interest in Calimmune, Inc. Calimmune provided no support for these studies, but is a subcontractor of the grant from CIRM.

## Supplementary Material

Additional file 1 **Distribution of simulated VIS data sets (400 and 1800 VIS) by chromosome**. The percentage of total VIS by chromosome in the full human X-linked ALD data set is shown in red. We sampled without replacement from the full human X-linked ALD data set to create ten simulated data sets per size for 12 sizes, ranging from 200 to 2000 VIS in intervals of 100 for 200-600 and intervals of 200 thereafter. The minimum and maximum percentages of VIS per chromosome for the 400 and 1800 VIS data sets are shown in the figure as blue and green bars, respectively. Click here for file

Additional file 2 **Convergence analysis of BCP method on full X-linked ALD data set**. Five start seeds were used to assess the convergence of the BCP results for run lengths of 500, 1000, 5000 and 10000 iterations. For each run length we plot the range of BCP-estimated z-scores (posterior means) for each bin versus the minimum z-score from the 5 differently seeded runs. In each plot there are 3091 points corresponding to the number of 1 Mb bins in the human data set. The dashed line indicates the z-score threshold of 422, where bins with z-scores to the right of this line were called hot-bins. The solid diagonal line indicates the lower bound for a bin's minimum z-score and range to achieve before it would be considered a hot-bin. Even for the short 500 iteration runs there were no cases where a bin was inconsistently called a hot-bin. However, the 500 and 1000 iteration runs (A-B) show that bins with minimum z-scores < 100 could have z-score differences of 45-65 for different start seeds. Z-score differences across start seeds becomes much smaller for bins with larger z-scores even for short 500 iteration runs. Among the 7 hot-bins corresponding to the 5 hot-spots reported in Table 3, a run length of 5000 iterations achieved posterior z-score estimates that had an SD of 0.007 or less. Based on these results we recommend a minimum run length of 5000 iterations. Click here for file

Additional file 3 **Differences between the z-threshold and BCP methods**. A comparison of hot-spot results between the BCP and z-threshold methods for the complete set of human X-linked ALD, X-linked CGD and X-linked SCID data sets (described in Table 1) shows four differences. The BCP method did not find hot-spots in the MLV acute infection data, whereas the z-threshold identified two (not shown). Also, two hot-spots that had z-scores near the cut-off threshold in the X-linked ALD patient 2 data at Chr 1 and Chr 3 were not found by the BCP method. The BCP method can miss some of the more minor hot-spots that the z-threshold method detects, but it detects fewer hot-spots in the acute infection data. Furthermore, analyses of additional data sets in our accompanying publication [24] have shown that in some cases the BCP method detects greater hot-spot coverage for strong signals (see rhesus macaque animal 2RC003 on Chr 14, below). Overall, these methods perform similarly, and we suggest running both for major data sets to check consistency of hot-spot results. Click here for file

Additional file 4 **CIS results for human data from SCIDX1, CGD, and X-linked ALD trials**. This plot is the CIS version of Figure 4A, showing the CIS hot-spots (indicated in red) on a genome level for all LV and MLV data sets. Grey indicates VIS that were not located in hot-spots. Data set names are as in Table 1. Click here for file

Additional file 5 **R software and tutorial for running the CIS, z-threshold and BCP hot-spot analyses**. This file contains R code and data sets to recreate the figures and table data presented in the paper. There are also instructions on how to change parameters to analyze other VIS data sets. See the 'README.txt' file for details. Click here for file
