# Supplementary figures and images for: Methodology and software to detect viral integration site hot-spots
Source: BMC Bioinformatics. 2011 Sep 14;12:367. doi: 10.1186/1471-2105-12-367 (PMC3203353; doi:10.1186/1471-2105-12-367)

% of VIS

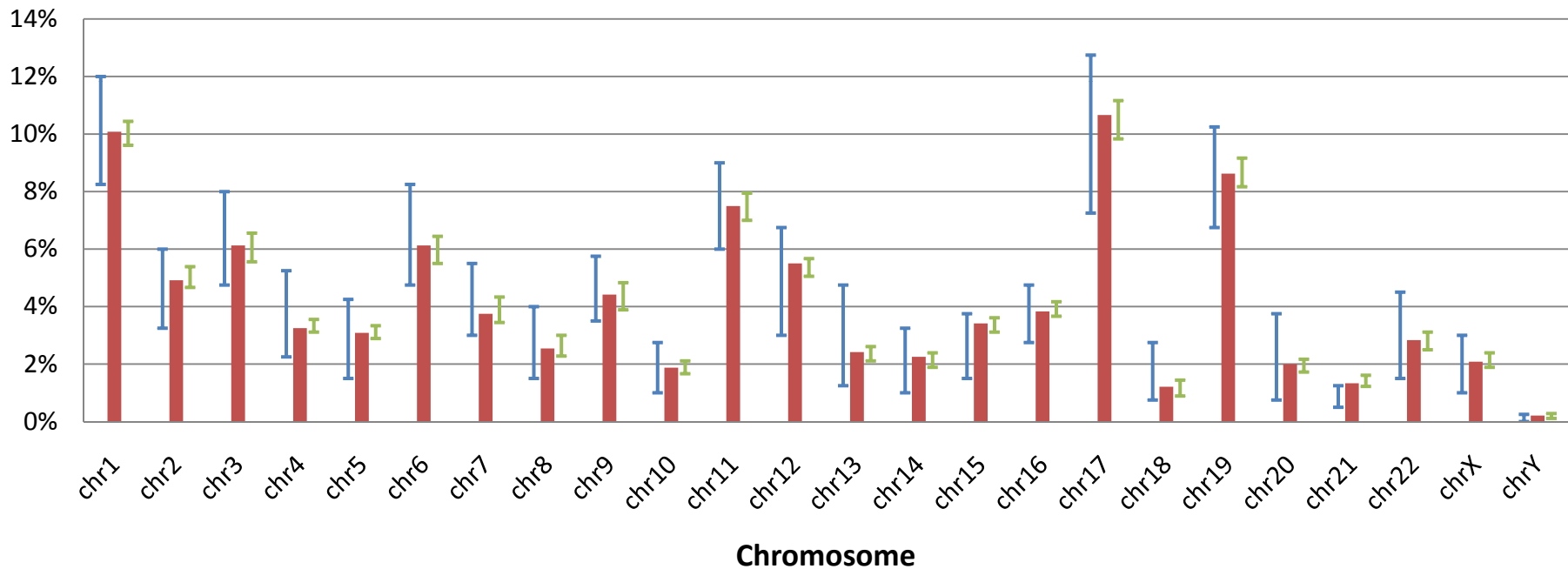

Supplement: Additional file 1 — Distribution of simulated VIS data sets (400 and 1800 VIS) by chromosome. The percentage of total VIS by chromosome in the full human X-linked ALD data set is shown in red. We sampled without replacement from the full human X-linked ALD data set to create ten simulated data sets per size for 12 sizes, ranging from 200 to 2000 VIS in intervals of 100 for 200-600 and intervals of 200 thereafter. The minimum and maximum percentages of VIS per chromosome for the 400 and 1800 VIS data sets are shown in the figure as blue and green bars, respectively. [file 1471-2105-12-367-S1.PDF]

**A. 500 Iterations**

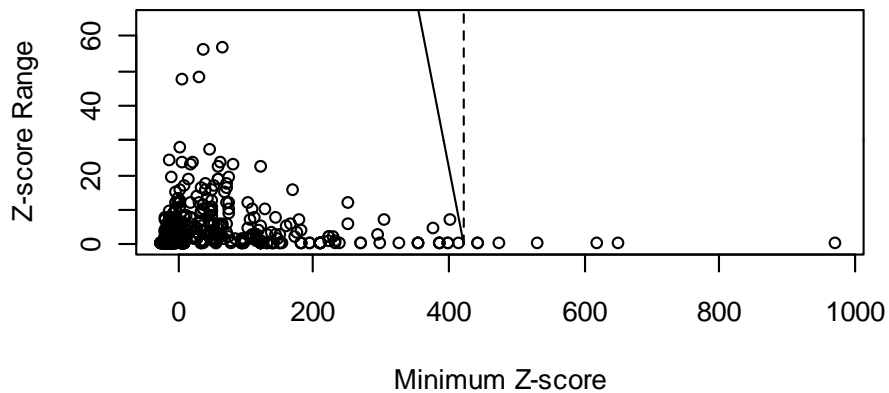

**B. 1000 Iterations**

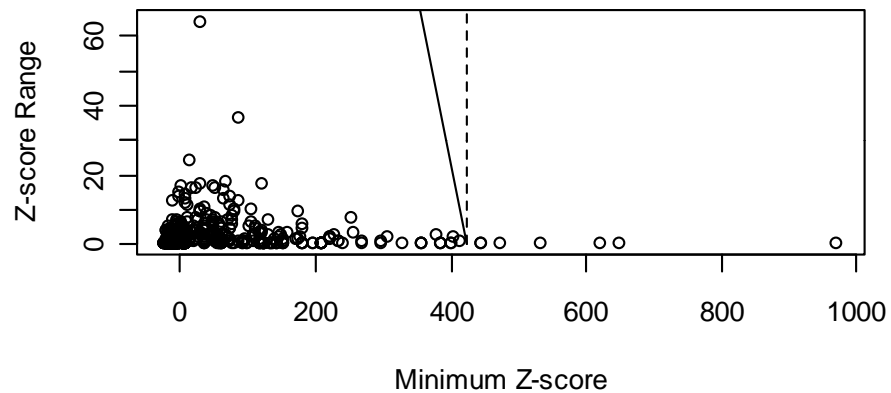

**C. 5000 Iterations**

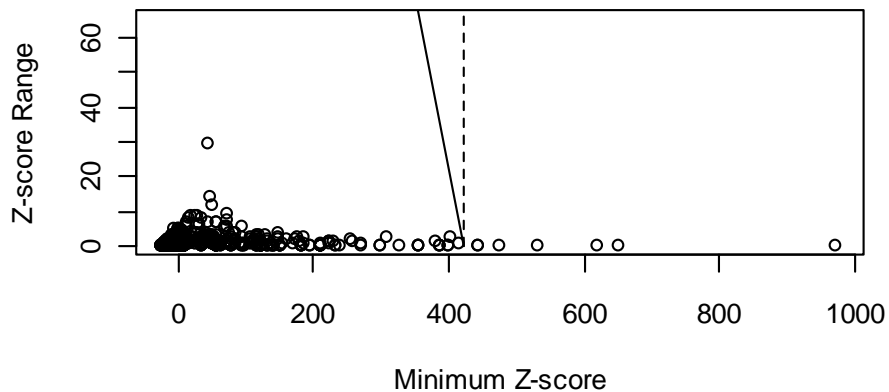

**D. 10000 Iterations**

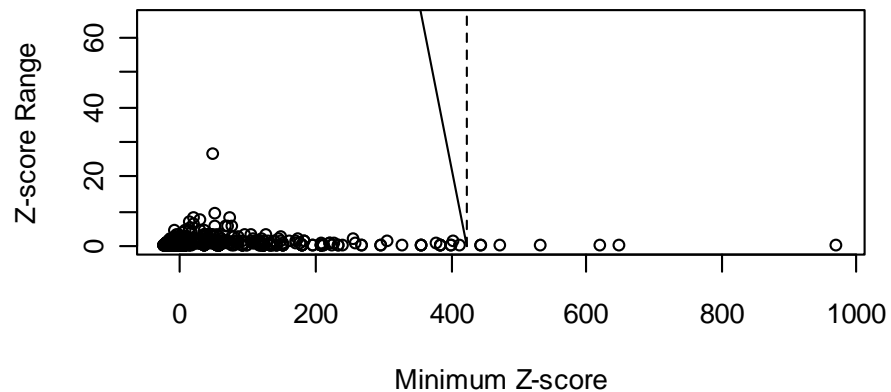

Supplement: Additional file 2 — Convergence analysis of BCP method on full X-linked ALD data set. Five start seeds were used to assess the convergence of the BCP results for run lengths of 500, 1000, 5000 and 10000 iterations. For each run length we plot the range of BCP-estimated z-scores (posterior means) for each bin versus the minimum z-score from the 5 differently seeded runs. In each plot there are 3091 points corresponding to the number of 1 Mb bins in the human data set. The dashed line indicates the z-score threshold of 422, where bins with z-scores to the right of this line were called hot-bins. The solid diagonal line indicates the lower bound for a bin's minimum z-score and range to achieve before it would be considered a hot-bin. Even for the short 500 iteration runs there were no cases where a bin was inconsistently called a hot-bin. However, the 500 and 1000 iteration runs (A-B) show that bins with minimum z-scores < 100 could have z-score differences of 45-65 for different start seeds. Z-score differences across start seeds becomes much smaller for bins with larger z-scores even for short 500 iteration runs. Among the 7 hot-bins corresponding to the 5 hot-spots reported in Table 3, a run length of 5000 iterations achieved posterior z-score estimates that had an SD of 0.007 or less. Based on these results we recommend a minimum run length of 5000 iterations. [file 1471-2105-12-367-S2.PDF]

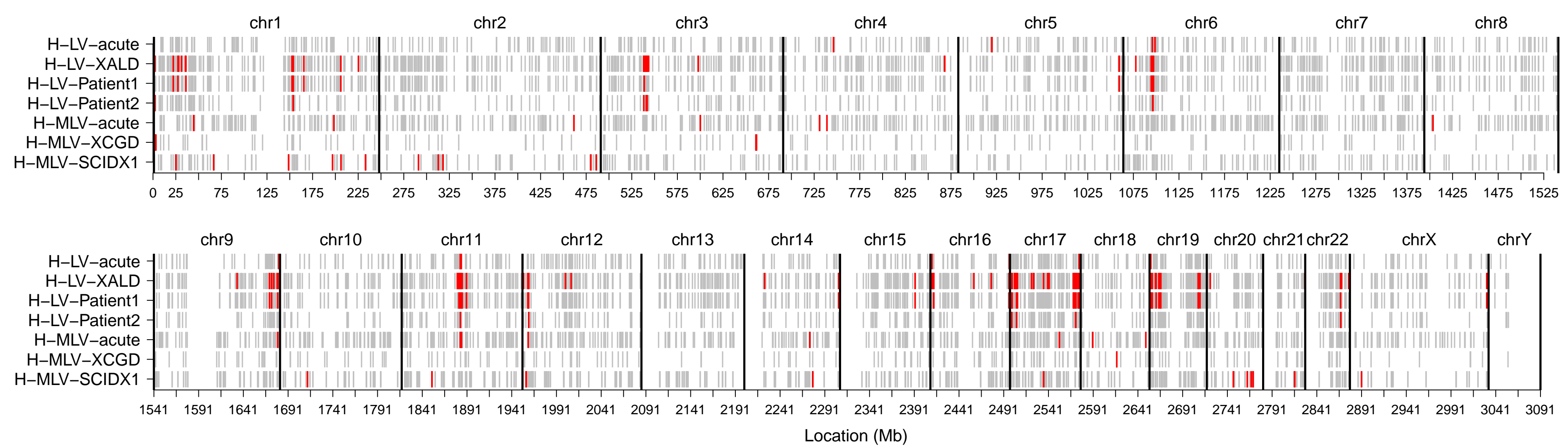

Supplement: Additional file 4 — CIS results for human data from SCIDX1, CGD, and X-linked ALD trials. This plot is the CIS version of Figure 4A, showing the CIS hot-spots (indicated in red) on a genome level for all LV and MLV data sets. Grey indicates VIS that were not located in hot-spots. Data set names are as in Table 1. [file 1471-2105-12-367-S4.PDF]
