# Supplementary material for: Methodology and software to detect viral integration site hot-spots
Source: BMC Bioinformatics. 2011 Sep 14;12:367. doi: 10.1186/1471-2105-12-367 (PMC3203353; doi:10.1186/1471-2105-12-367)

A. Bin threshold z-score hot-spot definition

B. BCP hot-spot definition

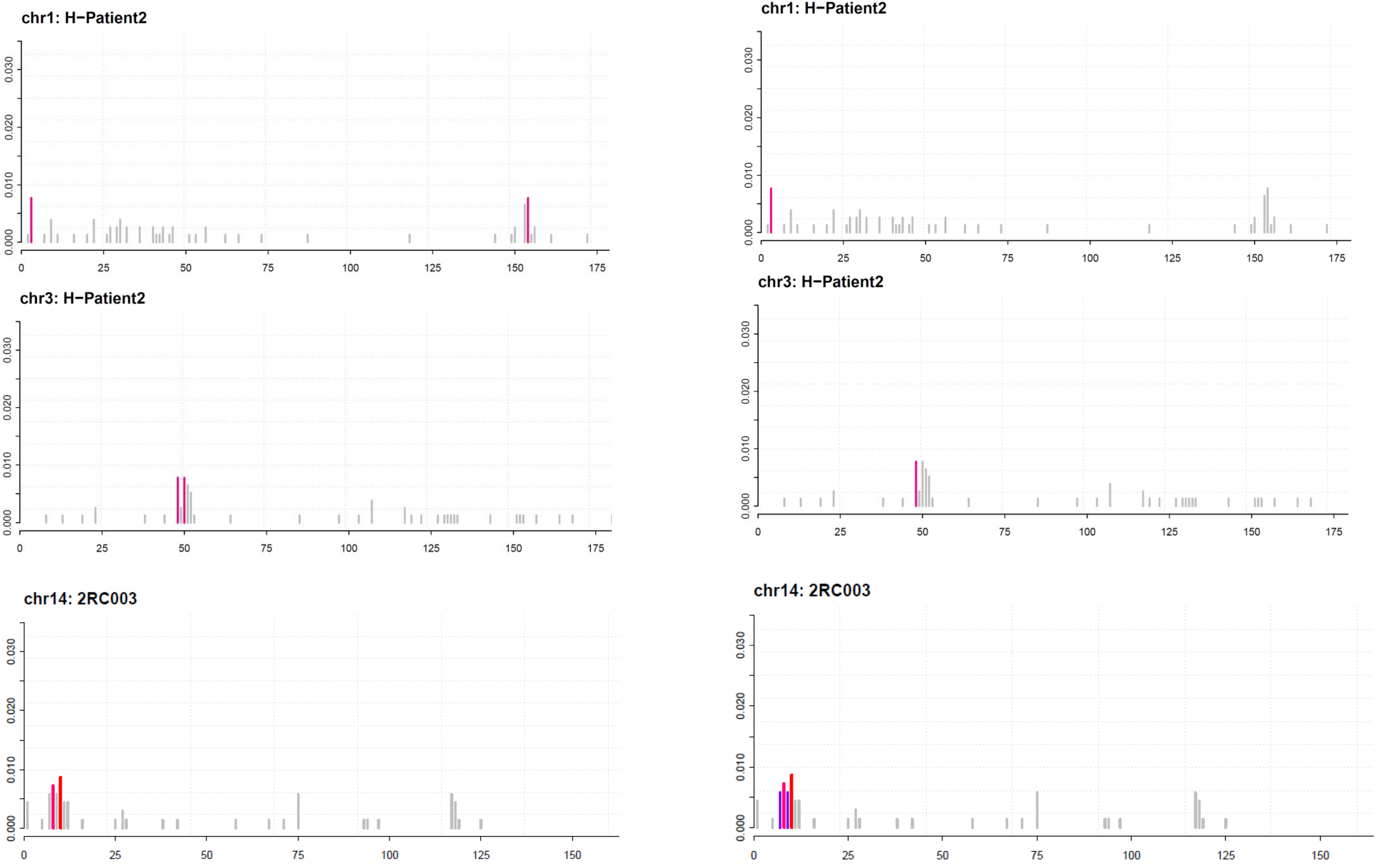

Supplement: Additional file 3 — Differences between the z-threshold and BCP methods. A comparison of hot-spot results between the BCP and z-threshold methods for the complete set of human X-linked ALD, X-linked CGD and X-linked SCID data sets (described in Table 1) shows four differences. The BCP method did not find hot-spots in the MLV acute infection data, whereas the z-threshold identified two (not shown). Also, two hot-spots that had z-scores near the cut-off threshold in the X-linked ALD patient 2 data at Chr 1 and Chr 3 were not found by the BCP method. The BCP method can miss some of the more minor hot-spots that the z-threshold method detects, but it detects fewer hot-spots in the acute infection data. Furthermore, analyses of additional data sets in our accompanying publication [24] have shown that in some cases the BCP method detects greater hot-spot coverage for strong signals (see rhesus macaque animal 2RC003 on Chr 14, below). Overall, these methods perform similarly, and we suggest running both for major data sets to check consistency of hot-spot results. [file 1471-2105-12-367-S3.PDF]
